# Feasibility and lessons learned on remote trial implementation from TestBoston, a fully remote, longitudinal, large-scale COVID-19 surveillance study

**DOI:** 10.1371/journal.pone.0269127

**Published:** 2022-06-03

**Authors:** Sarah Naz-McLean, Andy Kim, Andrew Zimmer, Hannah Laibinis, Jen Lapan, Paul Tyman, Jessica Hung, Christina Kelly, Himaja Nagireddy, Surya Narayanan-Pandit, Margaret McCarthy, Saee Ratnaparkhi, Henry Rutherford, Rajesh Patel, Scott Dryden-Peterson, Deborah T. Hung, Ann E. Woolley, Lisa A. Cosimi

**Affiliations:** 1 Division of Infectious Diseases, Brigham and Women’s Hospital, Boston, MA, United States of America; 2 Division of Epidemiology, University of Toronto Dalla Lana School of Public Health, Toronto, Canada; 3 Broad Institute of MIT and Harvard, Cambridge, MA, United States of America; 4 Division of General Internal Medicine and Primary Care, Brigham and Women’s Hospital, Boston, MA, United States of America; 5 Harvard Medical School, Boston, MA, United States of America; 6 Botswana Harvard AIDS Institute, Gaborone, Botswana; Freelance Consultant, Myanmar, MYANMAR

## Abstract

Longitudinal clinical studies traditionally require in-person study visits which are well documented to pose barriers to participation and contribute challenges to enrolling representative samples. Remote trial models may reduce barriers to research engagement, improve retention, and reach a more representative cohort. As remote trials become more common following the COVID-19 pandemic, a critical evaluation of this approach is imperative to optimize this paradigm shift in research. The TestBoston study was launched to understand prevalence and risk factors for COVID-19 infection in the greater Boston area through a fully remote home-testing model. Participants (adults, within 45 miles of Boston, MA) were recruited remotely from patient registries at Brigham and Women’s Hospital and the general public. Participants were provided with monthly and “on-demand” at-home SARS-CoV-2 RT-PCR and antibody testing using nasal swab and dried blood spot self-collection kits and electronic surveys to assess symptoms and risk factors for COVID-19 via an online dashboard. Between October 2020 and January 2021, we enrolled 10,289 participants reflective of Massachusetts census data. Mean age was 47 years (range 18–93), 5855 (56.9%) were assigned female sex at birth, 7181(69.8%) reported being White non-Hispanic, 952 (9.3%) Hispanic/Latinx, 925 (9.0%) Black, 889 (8.6%) Asian, and 342 (3.3%) other and/or more than one race. Lower initial enrollment among Black and Hispanic/Latinx individuals required an adaptive approach to recruitment, leveraging connections to the medical system, coupled with community partnerships to ensure a representative cohort. Longitudinal retention was higher among participants who were White non-Hispanic, older, working remotely, and with lower socioeconomic vulnerability. Implementation highlighted key differences in remote trial models as participants independently navigate study milestones, requiring a dedicated participant support team and robust technology platforms, to reduce barriers to enrollment, promote retention, and ensure scientific rigor and data quality. Remote clinical trial models offer tremendous potential to engage representative cohorts, scale biomedical research, and promote accessibility by reducing barriers common in traditional trial design. Barriers and burdens within remote trials may be experienced disproportionately across demographic groups. To maximize engagement and retention, researchers should prioritize intensive participant support, investment in technologic infrastructure and an adaptive approach to maximize engagement and retention.

## Introduction

Since its emergence in December 2019, the COVID-19 pandemic has struck a massive blow to world health and economic systems and exposed long-standing healthcare disparities with over 242 million confirmed infections and over 4.9 million deaths worldwide [1]. Accessibility and uptake of testing has varied between geographic locations and socio-economic groups, with many communities with the highest rates of COVID-19 simultaneously experiencing the lowest rates of testing [2, 3]. In October 2020, TestBoston, a longitudinal COVID-19 at-home testing study, was launched to understand the prevalence and risk factors for infection in the greater Boston area by providing access to SARS-CoV-2 viral and antibody testing with linkage to medical care and contact tracing. We hypothesized a fully remote model could reach a larger number of participants, while improving access to COVID-19 testing and biomedical research for underserved communities.

Disparities in clinical trial enrollment, particularly among Black and Hispanic/Latinx communities, are well-documented [4–7]. Barriers to participation range from structural factors including required time commitments, distance and transportation to clinical sites, language barriers, and hidden costs, to a legacy of fear and mistrust stemming from historical atrocities in biomedical research [6–13]. Remote models may provide greater efficiency, increased scale, wider geographic catchment areas, and the ability to reach a more representative population, including those unable or unwilling to travel for in-person study visits [5, 14–22].

In the United States, prior to the SARS-CoV-2 pandemic, hybrid models of research have practiced elements of remote data collection; however, fully remote, decentralized studies with remote enrollment and collection of biomedical samples are new to the landscape [23–25]. During the pandemic, clinical trials have faced unprecedented logistical barriers including social distancing protocols, restructuring of clinical sites to accommodate inpatient surges, participants’ fear of potential exposure during study visits, reduction of in-person research staff, and policies deeming study visits non-essential, necessitating adoption of remote methods to sustain research [5, 18, 26–30]. Rather than being constrained by these limitations, researchers have capitalized on the need to transform the landscape toward a more equitable and efficient future through implementation of remote study models [14, 17, 18, 21, 31, 32]. However, to date, there is minimal experience in defining best practices in this domain.

Here we present the methods used in launching and implementing a large-scale fully remote longitudinal at-home COVID-19 surveillance study. We highlight key successes, challenges, and critical lessons learned applicable to remote trial implementation regardless of disease domain.

## Methods

### Study design

Study eligibility included adults 18 years of age or older residing within a 45-mile radius of Boston, Massachusetts. Participants were recruited from the general public and through Mass General Brigham (MGB, formerly Partners Healthcare), a not-for-profit, integrated healthcare system with 14 affiliated hospitals via 1) “Physician invitation” for patients registered at MGB, who had seen any MGB physician within the previous 2 years, and included an introductory letter from the patient’s primary care or specialty provider; 2) “Direct invitation” for MGB patients who had previously opted-in to be directly contacted by MGB investigators about any research opportunities across the system; or 3) “Volunteer invitation” for individuals who signed up through an MGB-wide research recruitment website listing all future and ongoing studies (COVID-19 or otherwise) that are actively recruiting participants and are open to volunteers from the general public [33].Eligible individuals received an invitation letter containing a one-time code and instructions to visit the online study portal, enter the code, create an account, read and sign an online informed consent, input their mailing address, and respond to a brief demographic survey [34]. Completion of this process triggered a kit to be automatically sent to the participant. Interested individuals were encouraged to contact study staff to request assistance with the consent process, or to enroll by phone if unable to access the online portal. Study materials were translated into nine languages spoken in the geographical catchment area.

Extensive community outreach included consultation with state and local health departments to align study priorities, education sessions with MGB primary care and specialty clinics, outreach to community-based clinics and testing sites, press-releases, local news and radio segments, and partnering with local places of worship and community leaders to deliver general information about COVID-19 and answer questions from the community.

At enrollment, participants had the option to receive a one-time test kit, or to join the longitudinal cohort and receive monthly test kits for six months. Participants could request an additional “on-demand” kit at any time following exposure to COVID-19 or development of COVID-like symptoms. No financial incentives were provided for enrollment or kit completion.

**Fig 1:**
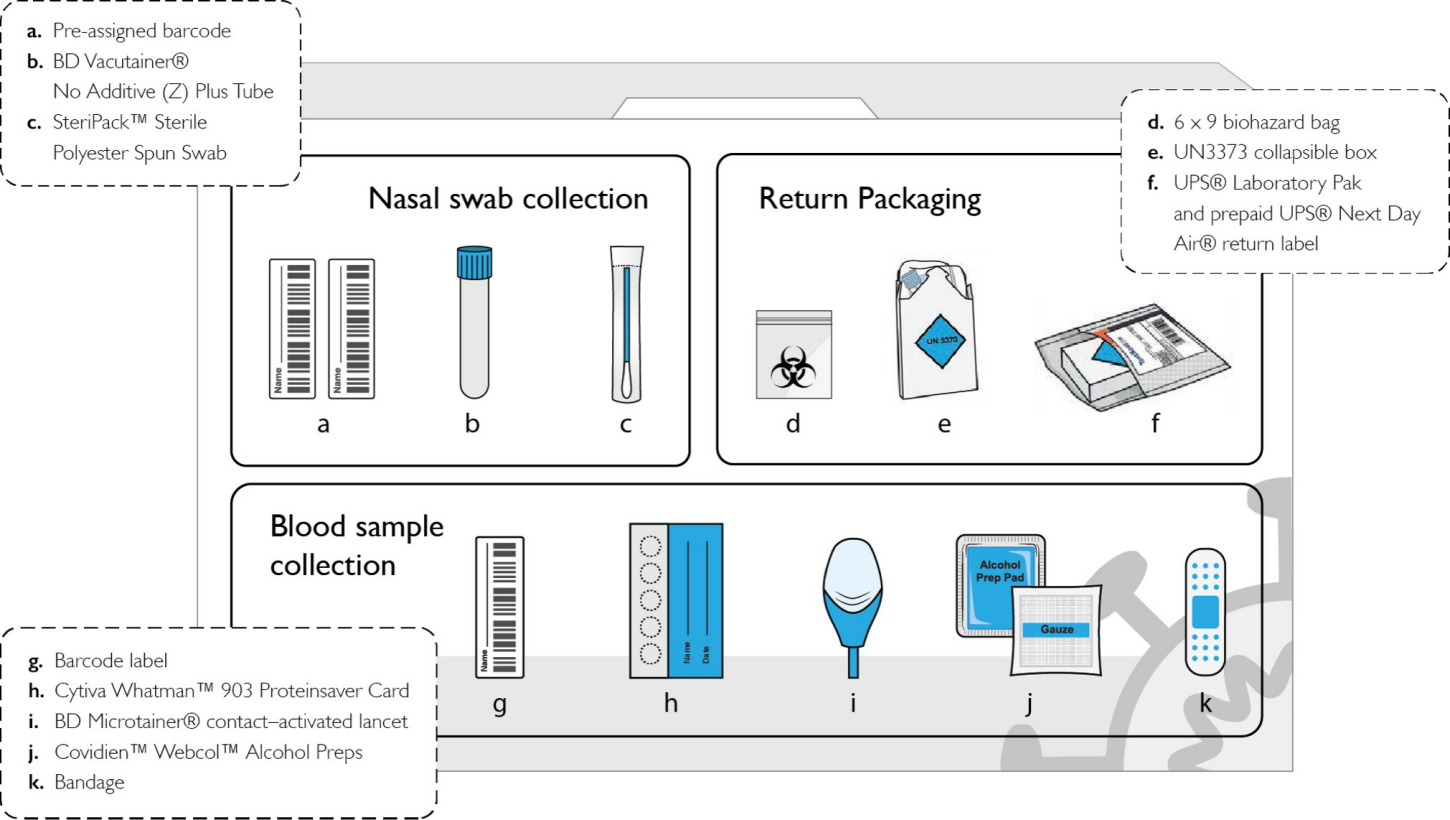
Schematic of TestBoston kit. The TestBoston COVID-19 sample collection kit consists of components for a self-collected anterior nasal swab for PCR testing, blood sample collection via finger prick and dried blood spot card for antibody testing, and return packaging.

**Fig 2:**
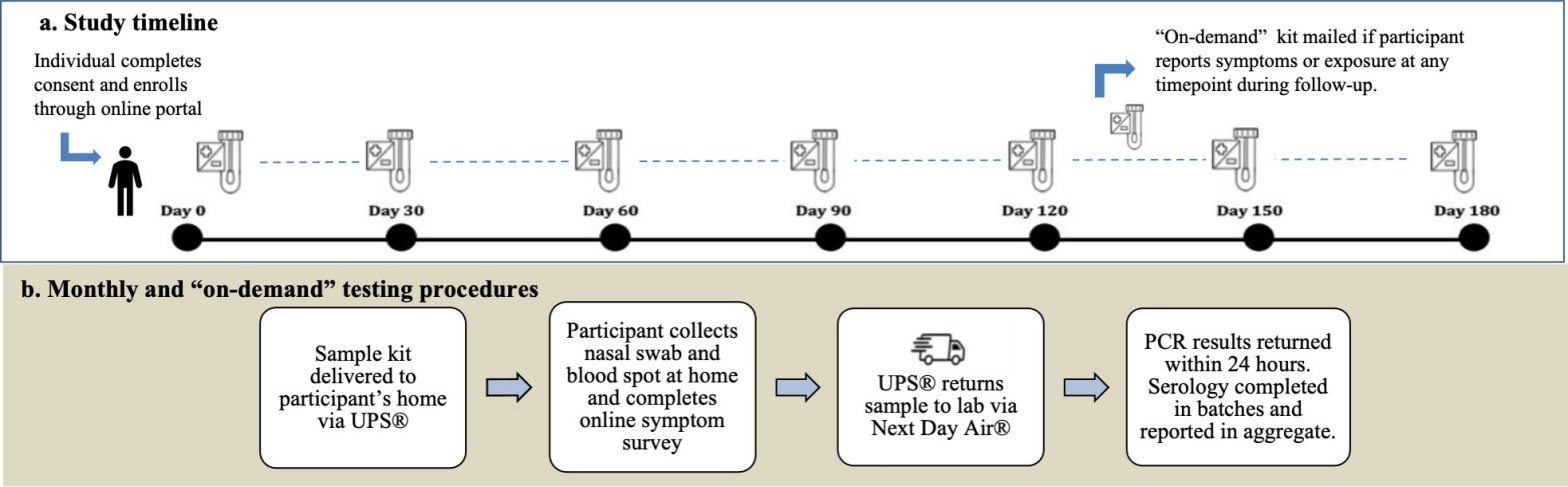
TestBoston procedures. The timeline for TestBoston participants (Part A) includes enrollment at time 0 and subsequent follow up for six calendar months. Routine monthly test kits are dispatched every 30 days, with the option to request “on-demand” kits at any timepoint if displaying symptoms consistent with COVID-19 or if exposed to COVID-19. Test kit procedures (Part B) include delivery, sample collection, return, and processing of test results.

Test kits were assembled and shipped from GBF Inc, High Point, NC and included an anterior nasal swab for reverse transcriptase–polymerase chain reaction (RT-PCR) testing and dried blood spot supplies for detection of SARS-CoV-2 antibody (Fig 1). When completing a kit, participants were directed to log in to their online portal to report recent exposures, new results of COVID-19 testing outside of the study, presence of symptoms concerning for COVID-19, and COVID-19 vaccination status (Fig 2).

Participants returned completed kits to the lab overnight via a United Parcel Service (UPS®) drop box or free home pickup. Upon arrival, kits were unboxed, reviewed for errors, then routed for high throughput RT-PCR testing in the COVID-19 Testing Program of Broad’s Clinical Research Sequencing Platform (CRSP). Samples were then inspected for quality control (S1 File), including a verification that participant name and barcode number for the returned sample matched kit distribution records. Viral RT-PCR results were delivered to participants’ online portals within 12–24 hours of sample receipt. Study staff notified individuals without computer access of their results by phone. Antibody results were reported in aggregate by zip code on the publicly available study website.

All positive RT-PCR results were reported directly to the Massachusetts Department of Public Health through the Massachusetts Epidemiologic Virtual Network (MAVEN), which triggered community contact tracing. A study physician contacted participants with positive results to offer post-test counseling and linkage to medical care through their primary care physician. Immediate referrals to either the emergency department or outpatient treatment of SARS-CoV-2, including monoclonal antibody treatments, were made based on current MGB guidelines [35].

### Data systems

Online enrollment, consent, longitudinal data collection, kit shipping, tracking and receiving, and return of RT-PCR results were supported by Pepper, an open-source software product built on the Google Cloud Platform and maintained by the Broad Institute Data Sciences Platform to configure and operate direct-to-participant studies [36]. Pepper provides Application Programming Interfaces (APIs) and user interfaces for participants, study team and logistical partners, utilizes 3rd party services, such as Auth0 for user authentication and authorization, SendGrid to distribute email communications to participants, and abides by all HIPAA security and breach rules. Participants were given a simple password-protected dashboard to complete study forms, request test kits, and view results. Data from Pepper was imported and supplemented with data from MGB medical records and stored in REDCap (Research Electronic Data Capture), a secure, web-based software platform designed to support data capture for research studies, hosted by MGB [37, 38].

### Data analysis

Longitudinal retention and engagement were measured based on number of kits returned, out of six, and time to kit return following delivery. We defined high level engagement as having completed five or more kits within 30 days of receipt; moderate engagement as 3 or 4 kits completed at any time point or 5 or more kits completed more than 30 days from receipt; and low engagement as having completed one or two kits at any time point. Multivariable Poisson regression was used with robust sandwich estimators to assess impact of the following baseline characteristics on level of engagement: sex, age (per 10 year increase), race and ethnicity, employment status (unemployed, employed remotely, or employed outside of home), and socioeconomic vulnerability as assessed by the Area Deprivation Index at census block group level [39]. Statistical analysis was performed in R, version 4.1.1 (R Foundation).

### Ethical considerations

This study was approved by the MGB Institutional Review Board.

## Results

### A multi-pronged approach leveraging links to the medical system enabled rapid, representative enrollment in absolute numbers, but exposed disparities in relative engagement

**Fig 3:**
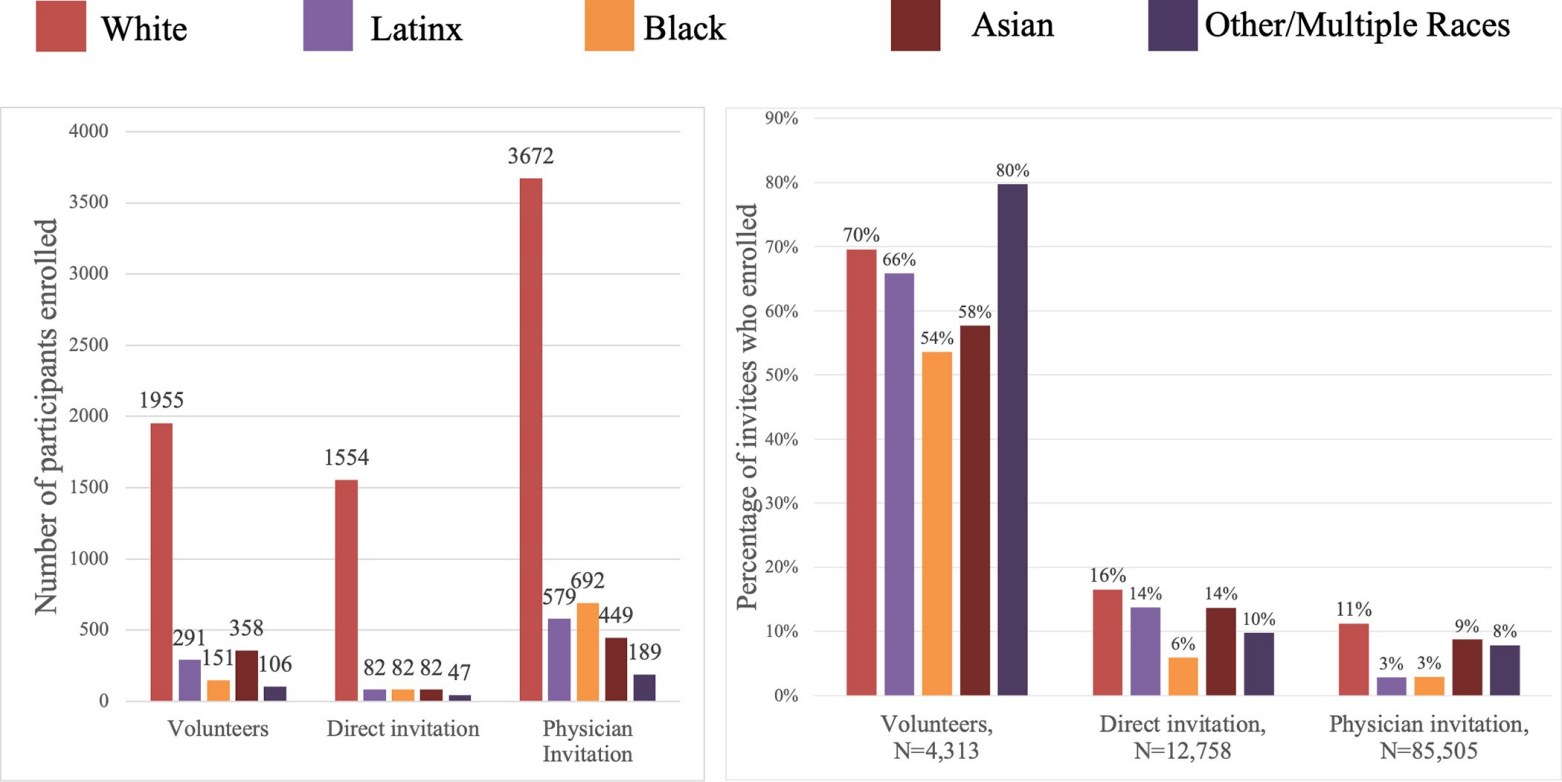
Enrollment by recruitment method. LEFT PANEL: Number of participants enrolled by recruitment method (Total enrolled cohort N = 10,289) RIGHT PANEL: Percent of invitees who accepted an invitation to enroll, by recruitment method (Total invited N = 102,576).

Between October 1, 2020 and February 2021, 102,576 people were invited to join the study, of which, 13,499 claimed their invitation code and started the registration and enrollment process and 10,289 (10.0%) fully enrolled and returned at least one of six possible kits. “Direct invitations” were sent to 12,758 MGB patients who previously opted-in to be contacted about research opportunities of whom 1,847 (14.5%) enrolled. “Physician invitations” were sent to 85,505 MGB patients of whom 5,581 (6.5%) enrolled. “Volunteer invitations” were sent to 4,313 individuals (with or without prior connection to MGB), of which 2861 (66.3%) enrolled (Fig 3, right panel). The connection to the hospital system was critical in allowing us to invite enough individuals to achieve a fully enrolled, demographically representative cohort (S2 File). This was particularly important for recruitment of Black and Hispanic/Latinx participants where regardless of invitation method, the enrollment rate was uniformly lower (Fig 3, left panel). In total, 692 (75.0%) of Black and 579 (60.8%) of Hispanic/Latinx participants enrolled through “physician invitation.” Daily monitoring of enrollment trends was critical to guide community outreach and adjustments in distribution of invitations resulting in enrichment of under-represented demographic groups invited to join the study to achieve a representative cohort (S2 File). Information sessions with local health departments, community-based clinics and community organizations served to increase direct referrals, make potential participants aware of the study, and validate its goals.

We observed high levels of retention across demographic groups with some differences in numbers of kits returned as a measure of engagement and retention. TestBoston was designed for participants in the longitudinal cohort to return six monthly kits with an anticipated 10% attrition rate per month resulting in a final engagement rate of ~50% after six months. Retention was slightly higher than expected with 5739 (55.8%) participants returning five or more kits within 30 days of receipt (Table 1). However, level of engagement and kit use behavior varied throughout the cohort and study period, mirroring the local COVID-19 surges and trends. We observed delays in participants completing kits due to travel, personal, or work obligations, as well as high volumes of kit use prior to holidays. The median number of days for return of monthly scheduled kits after delivery was six (IQR 2–13) compared to 3 days (IQR <1–8) for “on-demand” kits.

**Table 1:** Longitudinal retention & engagement based on kit completion.

	Demographics of total cohort		Prevalence of high, moderate, or low engagement by demographic group					
			High Engagement		Moderate Engagement		Low engagement	
	n	(% of total cohort)	n	(%)	n	(%)	n	(%)
**Overall engagement**	10289	(100%)	5739	(56%)	2582	(25%)	1968	(19%)
**Race/Ethnicity**								
White Non-Hispanic	7181	(70%)	4255	(59%)	1756	(24%)	1170	(16%)
Hispanic or Latinx	952	(9%)	419	(44%)	272	(29%)	261	(27%)
Black Non-Hispanic	925	(9%)	468	(51%)	235	(25%)	222	(24%)
Asian Non-Hispanic	889	(9%)	439	(49%)	226	(25%)	224	(25%)
Other/Multiple Races	342	(3%)	158	(46%)	93	(27%)	91	(27%)
**Age**								
18–29	1767	(17%)	712	(40%)	567	(32%)	488	(28%)
30–39	2309	(22%)	1048	(45%)	718	(31%)	543	(24%)
40–49	1828	(18%)	989	(54%)	468	(26%)	371	(20%)
50–59	1819	(18%)	1157	(64%)	382	(21%)	280	(15%)
60–69	1551	(15%)	1101	(71%)	268	(17%)	182	(12%)
70+	1015	(10%)	732	(72%)	179	(18%)	104	(10%)
**Sex assigned at birth**								
Female	5855	(57%)	3170	(54%)	1512	(26%)	1173	(20%)
Male	4434	(43%)	2569	(58%)	1070	(24%)	795	(18%)
**ADI quintile**								
1	233	(2%)	129	(55%)	49	(21%)	55	(24%)
2	818	(8%)	428	(52%)	197	(24%)	193	(24%)
3	1710	(17%)	896	(52%)	446	(26%)	368	(22%)
4	2767	(27%)	1576	(57%)	701	(25%)	490	(18%)
5	4742	(46%)	2710	(57%)	1188	(25%)	844	(18%)

Table 1 displays the overall demographic distribution of the TestBoston cohort, as well as the prevalence of engagement types by demographic group. High Engagement represents those individuals who completed 5 or more kits within 30 days of receipt. Moderate Engagement represents those who completed either 3 or 4 kits at any time point or completed 5 or more kits >30 days. Low engagement represents those who completed 1–2 kits at any time point.

In a multivariable model, there were modestly higher levels of engagement observed among participants who were White non-Hispanic (adjusted relative risk [aRR], 1.11 compared with non-White or Hispanic; 95% CI 1.09 to 1.13), older (aRR, 1.06 for each 10 year increase; 95% CI 1.06 to 1.07), had a lower neighborhood disadvantage (aRR, 1.01 per ADI quintile increase; 95% CI 1.00 to 1.02), and those working remotely (aRR, 1.03 compared with unemployed or students; 95% CI 1.01 to 1.05). No statistically significant differences were observed by sex or those working in-person (Table 2).

**Table 2:** Longitudinal retention & engagement based on kit completion.

Predictors	RR	(95% CI)	P_value_
White-Non Hispanic (vs all others)	1.11	(1.09–1.13)	P<0.001
Male (vs female)	0.99	(0.98–1.01)	0.37
Age (per 10 year increase)	1.06	(1.06–1.07)	P<0.001
ADI (per quintile decrease in disadvantage)	1.01	(1.00–1.02)	0.012
Employed outside home (vs unemployed, student, or missing)	0.99	(0.97–1.02)	0.578
Employed remote (vs unemployed, student, or missing)	1.03	(1.01–1.05)	0.008

Table 2 displays the relative risk of being in the “high engagement” category compared to low or moderate engagement by demographic variable. Multivariable Poisson regression was used with robust sandwich estimators to assess impact of the following baseline characteristics on level of engagement: sex, age (per 10 year increase), race and ethnicity, employment status (unemployed, employed remotely, or employed outside of home), and socioeconomic vulnerability as assessed by the Area Deprivation Index at census block group level

### Study implementation exposed key areas of participant burden unique to remote trials models requiring intensive staff support

Participants successfully returned 44,277 test kits, of which 95.7% of nasal swabs were satisfactory and resulted. Compared to in-person trials where participants travel to study sites and are guided through procedures, TestBoston participants were required to independently navigate participation, including online registration, consent, survey completion, self-directed sample collection and shipment. Participants highlighted burdens including time required to complete the multi-step sample collection process, in particular the dried blood spot card, and difficulties adhering to the same-day sample shipping protocol required to ensure arrival at the lab within the requisite timeframe for accurate testing.

**Fig 4:**
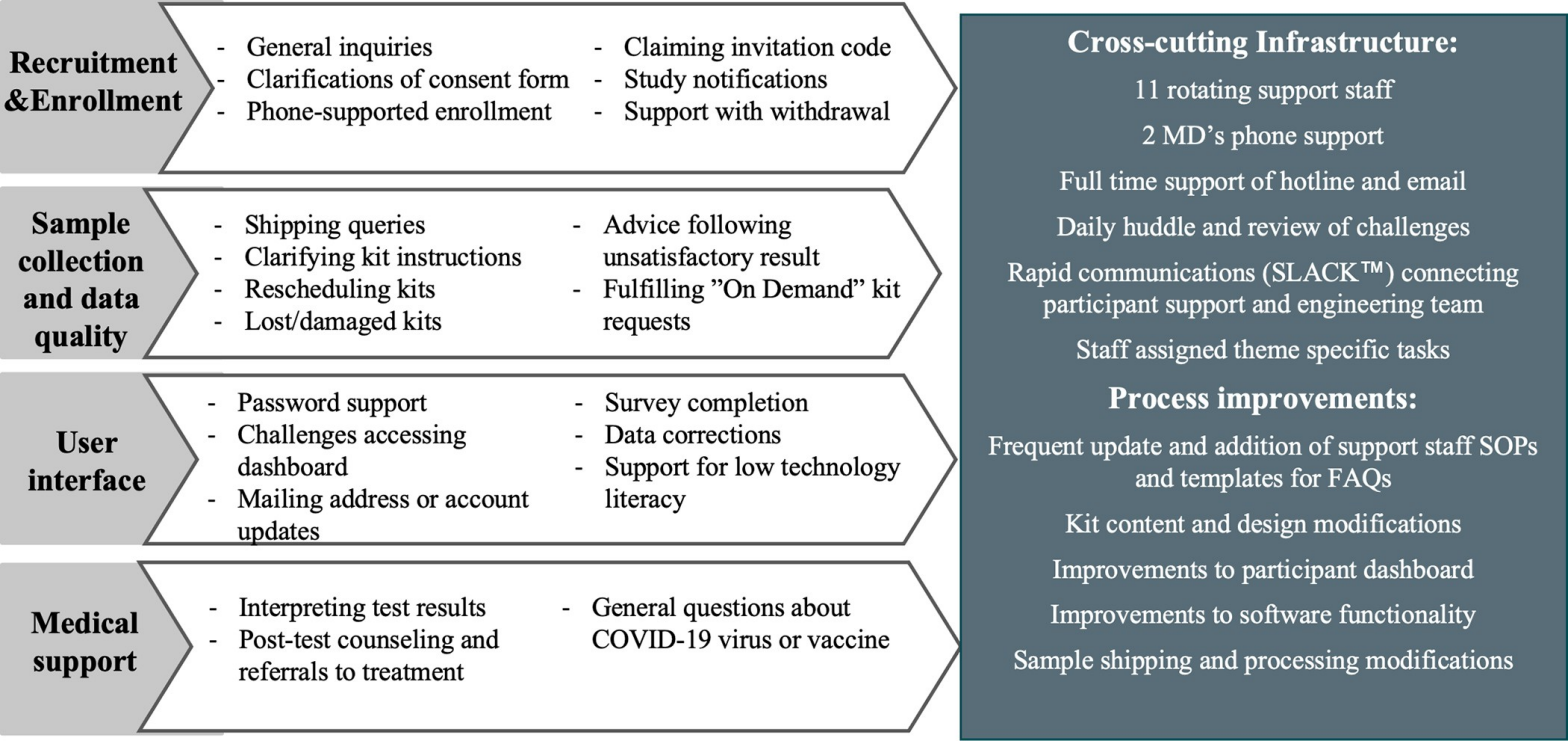
Participant support needs & cross-cutting infrastructure. Participant support needs (shown on the four left panels) fell into the following key domains: 1) Recruitment and enrollment, 2) sample collection and data quality, 3) user interface, and 4) medical support. As these needs emerged, the study team adopted numerous strategies to improve the participant experience and streamline operations (right panel).

While the remote and automated nature of the study design reduced many tasks that would have been performed by study staff in traditional in-person visits, the additional burdens experienced by participants led to higher than anticipated study staff support requirements. Throughout the study, participants sent 11,500 emails. While the quantity of hotline calls and voicemails was not tracked, eleven rotating support staff (one to two full-time staff members at any time) were dedicated solely to answering hotline calls Monday through Friday during business hours. Average encounters ranged from approximately 5 minutes or less for simple questions, to 30 minutes for those requiring more in-depth support, such as phone-assisted sample collection. Trends in participant support needs were categorized in four broad domains: recruitment & enrollment, sample collection, user interface, and medical support (Fig 4). We therefore assigned a dedicated team member to spearhead each thematic area. One of the most persistent needs was assisting participants in completing their monthly surveys. Over 12,300 (28%) returned kits did not have a completed symptom survey at time of sample collection, necessitating study staff follow-up. Intensive simultaneous support from six study team members was required to address high volume issues that arose, including uncontrollable, external events such as a worldwide outages of 3^rd^ party cloud services that impacted participant dashboard access, or nationwide holiday shipping delays that affected kit delivery. Finally, for participants with a positive COVID-19 diagnosis, physician support was critical in providing post-test counseling, assessing symptoms and risk profile, notifying the participant’s primary care physician, and referring for follow-up medical evaluation when needed.

### A robust information technology infrastructure coupled with participant-informed data review enabled an adaptive, iterative approach to support engagement and data quality

Technological infrastructure and information technology (IT) automation were imperative for TestBoston implementation, scalability, monitoring of individual participant progress through study milestones, and tracking macro level trends in real-time. During the three-month enrollment period, the mean daily enrollment was 83 participants (range 2–914) and the mean daily test kits ordered was 330 (range 17–1684). At full enrollment, a daily mean of 525 completed kits (range 210–984), each individually barcoded and tracked, was returned to the testing lab. Pepper acted as a centralized clearing house of data to manage and track each step of participant progress starting with participant-entered survey data, to data from 3^rd^ party systems reporting kit order fulfillment, delivery status, processing and testing within the lab, and automated return of test results. This system was essential, for example, in rapidly identifying unused kits, allowing the study team to send reminder emails, offer collection support, or order replacements kits when needed. Throughout the study 1567 kits were replaced for those lost in transit, damaged, missing components, or otherwise misplaced.

The integration of the technology infrastructure, software engineers and study staff in the form of rapid communications, and daily reviews of study status and frequently asked questions enabled us to routinely monitor data and integrate feedback obtained through participant-staff support encounters. This led to near constant review and improvement, including both immediate resolution of challenges for individual participants and more systemic operational and software changes (Fig 4). Standard operating procedures and template responses for over sixty frequently asked questions were modified in response to real-time data monitoring and participant challenges. Additionally, we were able to reduce the monthly unsatisfactory rates of returning nasal swabs for RT-PCR testing from 5.8% to 2.4% by instituting several processes. The most common reasons for failure of quality control included samples unsuitable for automated processing (typically related to viscosity or user error when affixing barcode labels), expired samples, and swab related errors (such as being upside down or broken) (S1 File). A dedicated study staff member reviewed daily unsatisfactory results and contacted participants to offer a re-test along with targeted advice for recollection based on the reason for rejection. Sample quality was further improved by more systemic changes as the study progressed, including revisions to the kit design to simplify collection instructions, and changes to the shipping and receiving infrastructure.

## Discussion

Recruitment, accrual, and retention are known challenges in biomedical research. We enrolled a cohort of over 10,000 individuals closely matching demographics of the greater Boston area with high levels of retention. Our model highlighted the critical role engaging physicians and leveraging connections to the medical system play in large-scale research recruitment in general, but specifically to ensure equitable representation. While the acceptance of an invitation was lower than among volunteers, the physician connection was critical in reaching demographic targets since the number of invitations extended through this avenue was twelve times higher. However, disparities we observed in enrollment rates also highlight ongoing barriers not alleviated by the convenience of remote models, such as fear or research mistrust, and underscores the need for additional work to achieve equitable and representative enrollment.

The relatively lower retention rates observed among participants who were Black, Hispanic/Latinx, Asian, younger, and of higher neighborhood vulnerability suggest barriers once enrolled in remote trials may also be experienced disproportionally across demographic groups. While remote trials may alleviate structural barriers by allowing flexibility based on personal availability, TestBoston participants highlighted work and family obligations, time, and scheduling constraints that conflicted with sample return, as significant barriers. Other potential gaps that have been described include technology access, literacy and privacy concerns that may be unaddressed, and at worst, exacerbated by remote models [14, 40–43]. We attempted to mitigate these factors by providing translation services, intense participant support, and participant-informed adaptations. However, additional research is needed to further understand these barriers and identify solutions, along with a commitment to a participant-partnered approach [14, 15, 20, 21].

The TestBoston model enabled fast, efficient enrollment and collection of longitudinal infectious disease surveillance data at the height of the pandemic. This would not have been feasible using an in-person approach, which would have required over 44,000 discrete study visits. The approach decreased certain burdens for staff including scheduling visits, collecting and processing samples, and for participants including travel time and costs, that occur with traditional in-person visits. However, with the onus on participants to independently adhere to study protocols, a robust level of remote support was critical to optimize the participant experience and ensure sample and data quality. These per-participant “transferred burdens” [20] experienced by study staff lessened over time and as the cohort grew, ultimately resulting in a highly efficient model at scale.

Our technology platform played an important role to enable the large volume of participants to self-report data and study staff to manage data, monitor participant progress, track kits as they pass through chain of custody, and respond to test results. This infrastructure was an important up-front investment that allowed us to rapidly reach a larger scale of participants more efficiently than would be possible through in-person data collection, but also required optimization in response to challenges in participant and staff usage, necessitating an ongoing investment over time.

The most critical implementation lesson was the need for an adaptive approach. This was necessary given the inability to fully a priori anticipate all real-world challenges encountered throughout a remote study during a pandemic. Unanticipated challenges arose due to scaling, uncontrollable third-party events, or because real-world participant behavior deviated unpredictably from ideal behavior. While impossible to prepare for every contingency, it was critical to adopt a strategy to rapidly receive feedback and adapt.

### Limitations

Translation of our methods to other studies must be considered within the context of the unprecedented circumstances of the COVID-19 pandemic. Enrollment was likely enhanced by the attraction of receiving at-home COVID-19 testing at a time when Boston was entering a second surge of infections and access to testing was still limited. COVID-19 created an environment that was paradoxically both amenable to certain innovations and resistant to typically straightforward operations. TestBoston benefited from high levels of political will and a shared urgency from institutional and community stakeholders which helped to overcome barriers and delays in implementing a fully remote study.

While a number of our findings, including required infrastructure, staffing support, participant challenges, and efforts to ensure sample and data integrity, may be broadly applicable to remote trial models regardless of disease domain, our findings related to recruitment and long-term engagement in remote clinical trials should be interpreted with caution given the short-term length of the study, and rapidly evolving context (including changes in COVID-19 epidemiology, availability of vaccines) which may have influenced interest in testing. Methods to optimize long-term participant engagement in remote trials will need continued attention.

While TestBoston succeeded in enrolling a demographically representative cohort based on Boston census data, there are shortcomings in using population as a benchmark for successful representation where a disease disproportionately impacts specific populations, in this case, Black and Hispanic/Latinx communities [44]. There was inherent tension in trying to study the disease in an unbiased way and, concurrently wanting to help communities most affected by COVID-19 by providing greater access to testing. Finally, though our approach achieved engagement of those traditionally neglected from research, including those unable or unwilling to travel to in-person study visits, most participants were affiliated with the hospital network, and may not equally represent those who do not have access to healthcare.

## Conclusions

As shown by the TestBoston model, the decentralization of clinical trials offers tremendous potential to disrupt the clinical trial landscape by reaching more representative cohorts and increasing scale, reducing per-participant time commitments for study staff, and promoting accessibility. Such studies must appreciate the different set of challenges created, compared to in-person studies; as the responsibility to complete study activities is transferred to enrolled individuals, sufficient investment in resources in the form of participant support and software infrastructure are needed to ease participant burdens. These unique challenges will likely be universal across remote trial study design regardless of research area. Researchers undertaking remote models must prioritize continuous learning from participants at all stages, observing real-world experiences to ensure this potentially paradigm-shifting model does not create new, different barriers to inclusion, but rather is a true opportunity for more representative research involvement.

## Supporting information

S1 File Sample quality control rejection criteria. 1A: Individual test result codes. When a sample is returned to the Broad Institute for processing and testing, it will be issued one of four possible results. Supplement 1A displays the result codes and associated definitions. 1B: Unsatisfactory/TNP (Test Not Performed) Reason Codes. Samples undergo rigorous quality control prior to testing, and samples that fail quality control are issued an “Unsatisfactory/TNP” result. Supplement 1B displays the unsatisfactory codes and corresponding definitions with the reasons for quality control failure.
(DOCX)

S2 File TestBoston cohort demographics compared to Boston census data. Supplement 2 displays the demographic distribution of the TestBoston cohort by race/ethnicity, compared to 2019 census data for the city of Boston.
(DOCX)
