# Supplementary material for: Feasibility and lessons learned on remote trial implementation from TestBoston, a fully remote, longitudinal, large-scale COVID-19 surveillance study
Source: PLoS One. 2022 Jun 3;17(6):e0269127. doi: 10.1371/journal.pone.0269127 (PMC9165767; doi:10.1371/journal.pone.0269127)
Supplement: S1 File — 1A: Individual test result codes. When a sample is returned to the Broad Institute for processing and testing, it will be issued one of four possible results. Supplement 1A displays the result codes and associated definitions. 1B: Unsatisfactory/TNP (Test Not Performed) Reason Codes. Samples undergo rigorous quality control prior to testing, and samples that fail quality control are issued an “Unsatisfactory/TNP” result. Supplement 1B displays the unsatisfactory codes and corresponding definitions with the reasons for quality control failure. (DOCX) [file pone.0269127.s001.docx]

**1A: Individual test result codes**

| **Result** | **Definition** |
| --- | --- |
| POSITIVE | Positive for detection of 2019-novel Coronavirus (2019-nCoV) by qRT-PCR. |
| NEGATIVE | 2019-novel Coronavirus (2019-nCoV) not detected by the qRT-PCR assay. Consider testing for other respiratory viruses or re-collecting for 2019-nCoV testing. Note: Optimum timing for peak viral levels during infections caused by 2019-nCoV have not been determined. Collection of multiple specimens from the same patient may be necessary to detect the virus. |
| INVALID | This specimen failed to produce a valid result. An Invalid result can mean that no nucleic acids (viral or human) were detected, or that the result otherwise did not meet our quality control specifications. Recommend re-collection of specimen. |
| INCONCLUSIVE | Inconclusive for SARS-CoV-2 by RT-PCR with one of the two viral probes (either N1 or N2) being detected while the other is not detected. Recommend re-collection of specimen. |
| UNSATISFACTORY/TNP | Test Not Performed. A new sample must be submitted to the laboratory in order to receive a confirmed test result. *See chart below for Unsat/TNP Reason Codes* |

**1B: Unsatisfactory/TNP (Test Not Performed) Reason Codes**

| UNSATISFACTORY_1 | Unsatisfactory for 2019-novel Coronavirus (2019-nCoV) testing by PCR: improper specimen transport medium. | Applies to samples submitted in media.  The media cannot be pipetted (too viscous). |
| --- | --- | --- |
| UNSATISFACTORY_2 | Unsatisfactory for 2019-novel Coronavirus (2019-nCoV) testing by PCR: inappropriate timing of collection relative to specimen receipt. Specimens must be received within 56 hours for dry collection, 72 hours for swabs in media. | Samples are received more than 56 hours after dry collection time, or 72 hours after collection time for swabs in media. |
| UNSATISFACTORY_3 | Unsatisfactory for 2019-novel Coronavirus (2019-nCoV) testing by PCR: sample tube unlabeled. | Sample tube is unlabeled and cannot be matched to an order. |
| UNSATISFACTORY_4 | Unsatisfactory for 2019-novel Coronavirus (2019-nCoV) testing by PCR: insufficient volume. | Applies to samples submitted in media. The tube is either empty or does not contain enough media volume to perform the test. |
| UNSATISFACTORY_5 | Unsatisfactory for 2019-novel Coronavirus (2019-nCoV) testing by PCR: tube sample data does not match the submission form. | This is used when the order and tube label do not match. |
| UNSATISFACTORY_6 | Unsatisfactory for 2019-novel Coronavirus (2019-nCoV) testing by PCR: laboratory incident. | This is used when a test can not be performed due to a laboratory error and the sample must be resubmitted. |
| UNSATISFACTORY_7 | Unsatisfactory for 2019-novel Coronavirus (2019-nCoV) testing by PCR: leaking/broken tube. | This is used for broken or leaking tubes. Very similar to reason #10, but this is used for samples submitted in media. |
| UNSATISFACTORY_8 | Unsatisfactory for 2019-novel Coronavirus (2019-nCoV) testing by PCR: sample not received. | This is used when we have an electronic or physical order in hand but do not receive a physical specimen. |
| UNSATISFACTORY_9 | Unsatisfactory for 2019-novel Coronavirus (2019-nCoV) testing by PCR: incomplete requisition. | This is used when required elements of the requisition are missing and we are unable to obtain the information before the sample expires, or if there is not enough information to know who to reach out to for the missing information. |
| UNSATISFACTORY_10 | Unsatisfactory for 2019-novel Coronavirus (2019-nCoV) testing by PCR: specimen tube received uncapped or broken. | This is used in cases where the sample arrives in an uncapped or broken tube. |
| UNSATISFACTORY_11 | Unsatisfactory for 2019-novel Coronavirus (2019-nCoV) testing by PCR: sample unsuitable for automated processing. | The first step in processing is to add media to the swab. After adding media, some samples are too viscous to process. This most often happens when the patient does not blow their nose before collecting the sample, resulting in excessive mucus. This reason may also be used if we cannot read the barcode on the tube. |
| UNSATISFACTORY_12 | Unsatisfactory for 2019-novel Coronavirus (2019-nCoV) testing by PCR: swab collection not to submission standards. | This is used when the swab is upside down in the tube, there are multiple swabs in the tube, or when there is no swab in the tube. |
| CANCELED | Testing canceled per submitter. | Canceled by ordering provider. |
