# Supplementary material for: Feasibility and lessons learned on remote trial implementation from TestBoston, a fully remote, longitudinal, large-scale COVID-19 surveillance study
Source: PLoS One. 2022 Jun 3;17(6):e0269127. doi: 10.1371/journal.pone.0269127 (PMC9165767; doi:10.1371/journal.pone.0269127)
Supplement: Supplementary file 2 [file pone.0269127.s002.docx]

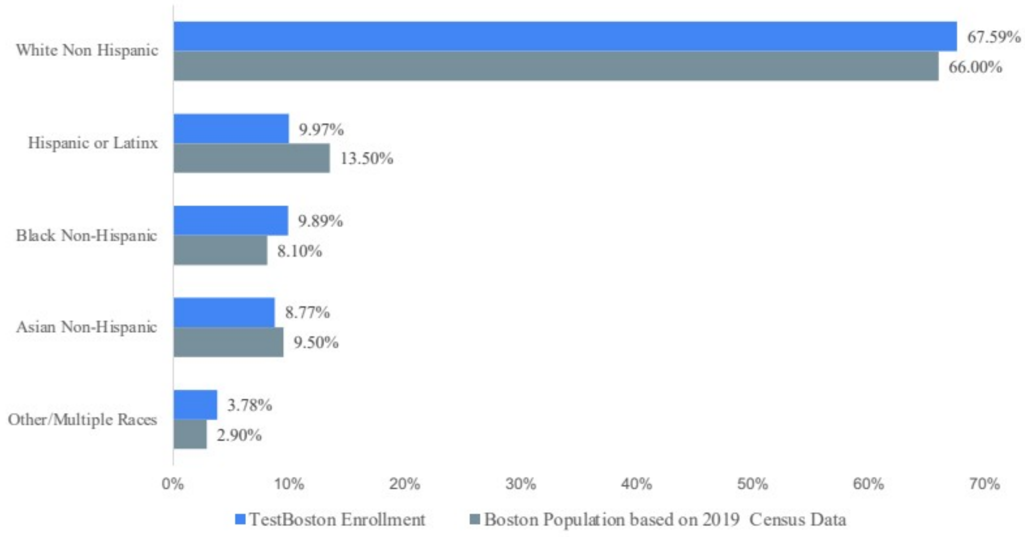
White Non Hispanic
67.59%
66.00%
Hispanic or Latinx
9.97%
13.50%
Black Non-Hispanic
9.89%
8.10%
Asian Non-Hispanic
8.77%
9.50%
Other/Multiple Races
3.78%
2.90%
0%
10%
20%
30%
40%
50%
60%
70%
TestBoston Enrollment
Boston Population based on 2019 Census Data
